# A reference gene set for sex pheromone biosynthesis and degradation genes from the diamondback moth, *Plutella xylostella*, based on genome and transcriptome digital gene expression analyses

**DOI:** 10.1186/s12864-017-3592-y

**Published:** 2017-03-01

**Authors:** Peng He, Yun-Fei Zhang, Duan-Yang Hong, Jun Wang, Xing-Liang Wang, Ling-Hua Zuo, Xian-Fu Tang, Wei-Ming Xu, Ming He

**Affiliations:** 1 State Key Laboratory Breeding Base of Green Pesticide and Agricultural Bioengineering, Key Laboratory of Green Pesticide and Agricultural Bioengineering, Ministry of Education, Guizhou University, Huaxi District, Guiyang, 550025 People’s Republic of China; 2 Biogas Institute of Ministry of Agriculture, Chengdu, 610041 People’s Republic of China; 3 The High Educational Key Laboratory of Guizhou Province for Natural Medicinal Pharmacology and Druggability, Guizhou Medical University, Huaxi university town, Guian new district, 550025 Guizhou, People’s Republic of China; 4 College of Plant Protection, Nanjing Agricultural University, Nanjing, 210095 People’s Republic of China; 5 Agriculture Economic and Rural Development, RENMIN University of China, Beijing, 100872 People’s Republic of China; 6 Guizhou Grass Jelly Biotechnology Company Limited, Chishui, Zhunyi 564700 People’s Republic of China

**Keywords:** Pheromone-biosynthesis enzymes, Pheromone-degrading enzymes, Detoxification, Carboxyl/Cholinesterase, Aldehyde oxidase, Fatty acyl reductase, Desaturase

## Abstract

**Background:**

Female moths synthesize species-specific sex pheromone components and release them to attract male moths, which depend on precise sex pheromone chemosensory system to locate females. Two types of genes involved in the sex pheromone biosynthesis and degradation pathways play essential roles in this important moth behavior. To understand the function of genes in the sex pheromone pathway, this study investigated the genome-wide and digital gene expression of sex pheromone biosynthesis and degradation genes in various adult tissues in the diamondback moth (DBM), *Plutella xylostella*, which is a notorious vegetable pest worldwide.

**Results:**

A massive transcriptome data (at least 39.04 Gb) was generated by sequencing 6 adult tissues including male antennae, female antennae, heads, legs, abdomen and female pheromone glands from DBM by using Illumina 4000 next-generation sequencing and mapping to a published DBM genome. Bioinformatics analysis yielded a total of 89,332 unigenes among which 87 transcripts were putatively related to seven gene families in the sex pheromone biosynthesis pathway. Among these, seven [two desaturases (DES), three fatty acyl-CoA reductases (FAR) one acetyltransferase (ACT) and one alcohol dehydrogenase (AD)] were mainly expressed in the pheromone glands with likely function in the three essential sex pheromone biosynthesis steps: desaturation, reduction, and esterification. We also identified 210 odorant-degradation related genes (including sex pheromone-degradation related genes) from seven major enzyme groups. Among these genes, 100 genes are new identified and two aldehyde oxidases (AOXs), one aldehyde dehydrogenase (ALDH), five carboxyl/cholinesterases (CCEs), five UDP-glycosyltransferases (UGTs), eight cytochrome P450 (CYP) and three glutathione S-transferases (GSTs) displayed more robust expression in the antennae, and thus are proposed to participate in the degradation of sex pheromone components and plant volatiles.

**Conclusions:**

To date, this is the most comprehensive gene data set of sex pheromone biosynthesis and degradation enzyme related genes in DBM created by genome- and transcriptome-wide identification, characterization and expression profiling. Our findings provide a basis to better understand the function of genes with tissue enriched expression. The results also provide information on the genes involved in sex pheromone biosynthesis and degradation, and may be useful to identify potential gene targets for pest control strategies by disrupting the insect-insect communication using pheromone-based behavioral antagonists.

**Electronic supplementary material:**

The online version of this article (doi:10.1186/s12864-017-3592-y) contains supplementary material, which is available to authorized users.

## Background

In moths, reproductive isolation relies heavily on mature female adults producing and releasing species-specific sex pheromone components to attract conspecific males. Adult male antennae on the other hand specifically perceive these sex pheromone components from a distance resulting in the successful location of the female to initiate and complete mating behavior [1]. In lepidopterans, type I sex pheromone components are fatty acid derivatives with 0–4 double bond containing carbon chains of varying lengths (C10-C18) and an oxygenated functional group that can be an aldehyde, alcohol or an acetate ester. Type II sex pheromone components are hydrocarbons or epoxides [2, 3]. In female moths, the type I sex pheromones are synthesized *de novo* and the genes involved in the biosynthesis are regulated in a specialized manner in their sex pheromone glands (PGs). Sex pheromone biosynthesis employs a modified fatty-acid biosynthesis pathway that includes various processes such as acetylation, desaturation, chain shortening, reduction, and oxidation, either separately or in combination [2, 4].

Type I sex pheromone biosynthesis pathway in moths initiates in PG by the release of pheromone biosynthesis activating neuropeptide (PBAN) from the subesophageal ganglion, which then circulates unbound to the PG where it binds to the pheromone biosynthesis activating neuropeptide receptor (PBANr) [5]. The resulting signal, then triggers several enzyme pathways starting with acetyl-CoA carboxylase (ACC) that catalyzes the conversion of acetyl-CoA to malonyl-CoA and finally resulting in the synthesis of pheromone precursors (mostly 14, 16 or 18 carbon saturated fatty acids) [4]. Later, double bonds are introduced into the pheromone precursors at specific positions (Δ5 [6], Δ6 [7], Δ9 [8–12], Δ10 [13, 14], Δ11 [9, 15, 16] and Δ14 [17, 18]) by different desaturases (DESs), and the chain is shortened by a modified β-oxidation pathway [19]. Finally, the unsaturated pheromone precursors are modified to form various functional groups such as aldehyde, alcohol or acetate ester, by three enzymes, aldehyde reductase, fatty acyl reductase and acetyltransferase, respectively [2, 20–22]. Some of these enzymes have been shown to have substrate specificity. For example, pgFARs from *Spodoptera exigua* have substrate preference for C14 and C16 fatty acids [23].

Males on the other hand, have evolved a sensitive antennal olfaction system to trace even very low amounts of sex pheromone components released from the female located at a long distance [24]. These sex pheromone components enter the male antennal pores and are transported by pheromone binding proteins (PBPs) [25–27] through the antennal sensillum, and activate membrane-bound pheromone receptors (PRs) [28–30]. After PR activation, the pheromone molecules must be rapidly degraded to release and refresh the PR. Depending on the functional group on a pheromone, whether an aldehyde, alcohol or ester, degradation of pheromones involves specific enzymes in the pheromone degradation pathway. Aldehyde oxidase (AOX) is an antennal enzyme that catalyzes the oxidation of aldehyde sex pheromones to carboxylic acids, which were biochemically characterized in the antennae of *Manduca sexta* [31]*, Antheraea polyphemus* and *Bombyx mori* [32]. Thus far, only one insect AOX (AtraAOX2) from *Amyelois transitella* has been functionally characterized to hydrolyze aldehyde sex pheromone components and some plant volatiles in vitro [33]. However, a number of putative AOX genes have been reported in lepidopterans at the nucleic acid level [34–36]. Aldehyde-degradation involves the NAD(P)^−^ dependent aldehyde dehydrogenase (ALDH) and AR enzymes that convert sex pheromones to their corresponding acids and alcohols, respectively [37–39]. Antennal carboxyl/cholinesterase (CCE) hydrolyzes ester sex pheromone components. In previous studies, we functionally characterized five carboxyl/cholinesterases (CXEs) belonging to the CCE family from *S. exigua* and *Spodoptera litura*; in vitro expressed SexiCXE10 specifically degraded ester plant volatiles and three other CCEs (SexiCXE4, SexiCXE13 and SlitCXE13) had dual roles, i.e., they hydrolyzed sex pheromone components as well as plant volatiles [40–43]. Other members of the multi-function enzyme families include CYPs [44, 45], UGTs [46] and GSTs [47], which are also considered as odorant-degrading enzymes (ODEs) or pheromone-degrading enzymes (PDEs).

The diamondback moth (DBM), *Plutella xylostella* (Lepidoptera: Plutellidae), is a notorious pest of cruciferous vegetables worldwide. The major known pheromone components extracted from female DBM moth PGs include three different functional groups that were type I sex pheromone components; (Z)-11-hexadecenal [Z11-16:Ald] [48], (Z)-11-hexadecenyl acetate [Z11-16:Ac] [49–52] and (Z)-11-hexadecenol [Z11-16:OH] [49–52]. These studies also revealed that both female and male DBM need precise regulation of sex pheromone biosynthesis that facilitates their communication through the various structural components. In recent years, sex pheromone reception has been studied extensively because of its potential use as green targets for environmentally friendly pest control measures. Male DBMs have PBPs that are designed for sex pheromone reception. Thus far, three PBPs have been cloned and characterized in *P. xylostella*. These PBPs robustly bound all three sex pheromone components (Z11-16:Ald, Z11-16:OH and Z11-16) [53] thereby enhancing the electrophysiological responses of DBM to sex pheromone components in vitro [54]. In the recently sequenced DBM genome [54], two gene sequences likely to be odorant/pheromone-degrading enzymes such as CYPs [55] and GSTs [56] were identified. However, the tissue specific expression of these genes are not known. To identify additional genes involved in the DBM pheromone biosynthesis and degradation, in this study, we combined transcriptome based digital gene expression (DGE) and genome mapping. By using these methods, we identified a total of 299 genes belonging to 14 gene families potentially involved in the sex pheromone biosynthesis and degradation pathways. The tissue specific expression pattern of these genes was also confirmed qPCR.

## Results and discussion

### Overview of the transcriptomes

**Fig. 1:**
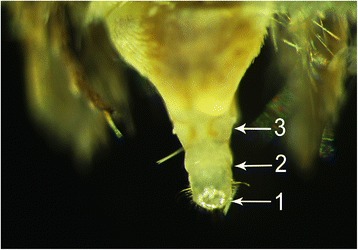
Dissection of *P. xylostella* female sex pheromone glands. The pheromone glands from female *P. xylostella* were squeezed out from the abdomen using forceps (the gland is similarly inflated when the female calls). The abdomen of *P. xylostella* was cut at the sclerotized cuticle from the 8^th^ abdominal segment, and the sclerotized cuticle was removed before immersing the glands in liquid nitrogen. **1:** Sclerotized ovipositor valves; **2:** Pheromone gland; **3:** Sclerotized cuticle that was removed

Six transcriptomes from male antennae (m_Ant), female antennae (f_Ant), legs (L), heads (H), abdomens (AB) and female pheromone glands (PG) were sequenced using the Illumina HiSeq 4000 platform (Illumina, Tianjin, China) and assembled with Trinity (version r20140413p1). To obtain an accurate expression pattern, we removed as much of the ovipositor as possible from the females due to its adherence to the pheromone glands (Fig. 1). Illumina sequencing yielded 5.96–6.84 giga bases (Gb) for each of the six transcriptomes. We first mapped the transcriptome reads to the DBM reference genome (Genome assembly version 2, http://iae.fafu.edu.cn/DBM/) (Table 1) and obtained the following mapping rates that were favourable for annotation: 42.29% (legs), 39.6% (male antennae), 38.65% (female antennae), 42.27% (heads), 43.7% (pheromone glands) and 41.65% (abdomens). Then, we supplemented the original genome annotation file (18,105 genes Genome assembly version 2, http://iae.fafu.edu.cn/DBM/) and found 3,839 novel genes (Additional file 1: Table S1). Further to obtain more unigenes, we also performed a *de novo* assembly of the six transcriptomes that yielded unigenes with a median length of 334 nt with the longest unigene being 28,680 nt in length. Finally, these reads were assembled into 125,385 transcripts and 89,332 unigenes, with N50 lengths of 1,831 and 1,499 nt, respectively (Table 2).

**Table 1:** Summary of *P. xylostella* transcriptome mapping to the reference genome

Sample_name	L	M_Ant	F_Ant	H	PG	AB
Total reads	53439024	54660234	50948026	51117268	47655468	54606672
Total mapped	22601392 (42.29%)	21647440 (39.6%)	19691333 (38.65%)	21606597 (42.27%)	20826116 (43.7%)	22742109 (41.65%)
Multiple mapped	2634934 (4.93%)	2802997 (5.13%)	2425347 (4.76%)	2059342 (4.03%)	2283645 (4.79%)	2736184 (5.01%)
Uniquely mapped	19966458 (37.36%)	18844443 (34.48%)	17265986 (33.89%)	19547255 (38.24%)	18542471 (38.91%)	20005925 (36.64%)
Read-1	10216135 (19.12%)	9618895 (17.6%)	8799050 (17.27%)	9995946 (19.55%)	9468501 (19.87%)	10206183 (18.69%)
Read-2	9750323 (18.25%)	9225548 (16.88%)	8466936 (16.62%)	9551309 (18.69%)	9073970 (19.04%)	9799742 (17.95%)
Reads map to ‘ + ’	9980188 (18.68%)	9391804 (17.18%)	8600732 (16.88%)	9758204 (19.09%)	9242300 (19.39%)	9976030 (18.27%)
Reads map to ‘-’	9986270 (18.69%)	9452639 (17.29%)	8665254 (17.01%)	9789051 (19.15%)	9300171 (19.52%)	10029895 (18.37%)
Non-splice reads	13506179 (25.27%)	12550858 (22.96%)	11270056 (22.12%)	10945264 (21.41%)	12244204 (25.69%)	13297124 (24.35%)
Splice reads	6460279 (12.09%)	6293585 (11.51%)	5995930 (11.77%)	8601991 (16.83%)	6298267 (13.22%)	6708801 (12.29%)

**Table 2:** Summary of *P. xylostella de novo* transcriptome assembly

Tissues	F_Ant	M_Ant	L	H	AB	PG
Total size (Gb)	6.36	6.84	6.68	6.38	5.96	6.82
GC content	47.26	45.98	47.46	54.59	47.77	45.01
Number of transcripts	125,385					
Total unigene count	89,332					
Genes with homologues in NR	26,657					
Total transcript nucleotides	113,082,390					
Total unigene nucleotides	65,915,278					
N50 transcript length	1831 nt					
N50 unigene length	1499 nt					
Longest unigene length	28,680 nt					
Median unigene length	334 nt					

BLASTx searches of all 89,332 unigenes showed that 29.84% were homologous to proteins in several insect genome databases (*Bombyx mori, Danaus plexippus, Acyrthosiphon pisum, Anopheles gambiae, Apis mellifera, Drosophila melanogaster, Tribolium castaneum, Lucilia cuprina, Rhodnius prolixus* and *Solenopsis invicta*, http://ensemblgenomes.org/info/genomes) with a cut-off E-value of 10^−5^. The highest homology (31.2%) was with *B. mori* sequences, followed by sequences from *D. plexippus* (27.8%), *A. gambiae* (12.6%), *D. melanogaster* (9.7%), and *T. castaneum* (5.3%) (Additional file 2: Figure S1a).

Then, we used Blast2GO to annotate unigenes into functional groups based on Gene ontology (GO). The GO annotations were used to classify the transcripts into functional groups according to specific GO categories. In the molecular function category, the genes expressed were mostly enriched for catalytic activity (e.g., hydrolase and oxidoreductase) and binding (e.g., nucleotide, ion, and odorant binding). In the biological process category, the most common were the cellular and metabolic processes. In the cellular component category, the most represented were the following two terms: cell (GO:0005623) and cell part (GO:0044464) (Additional file 2: Figure S1b).

**Fig. 2:**
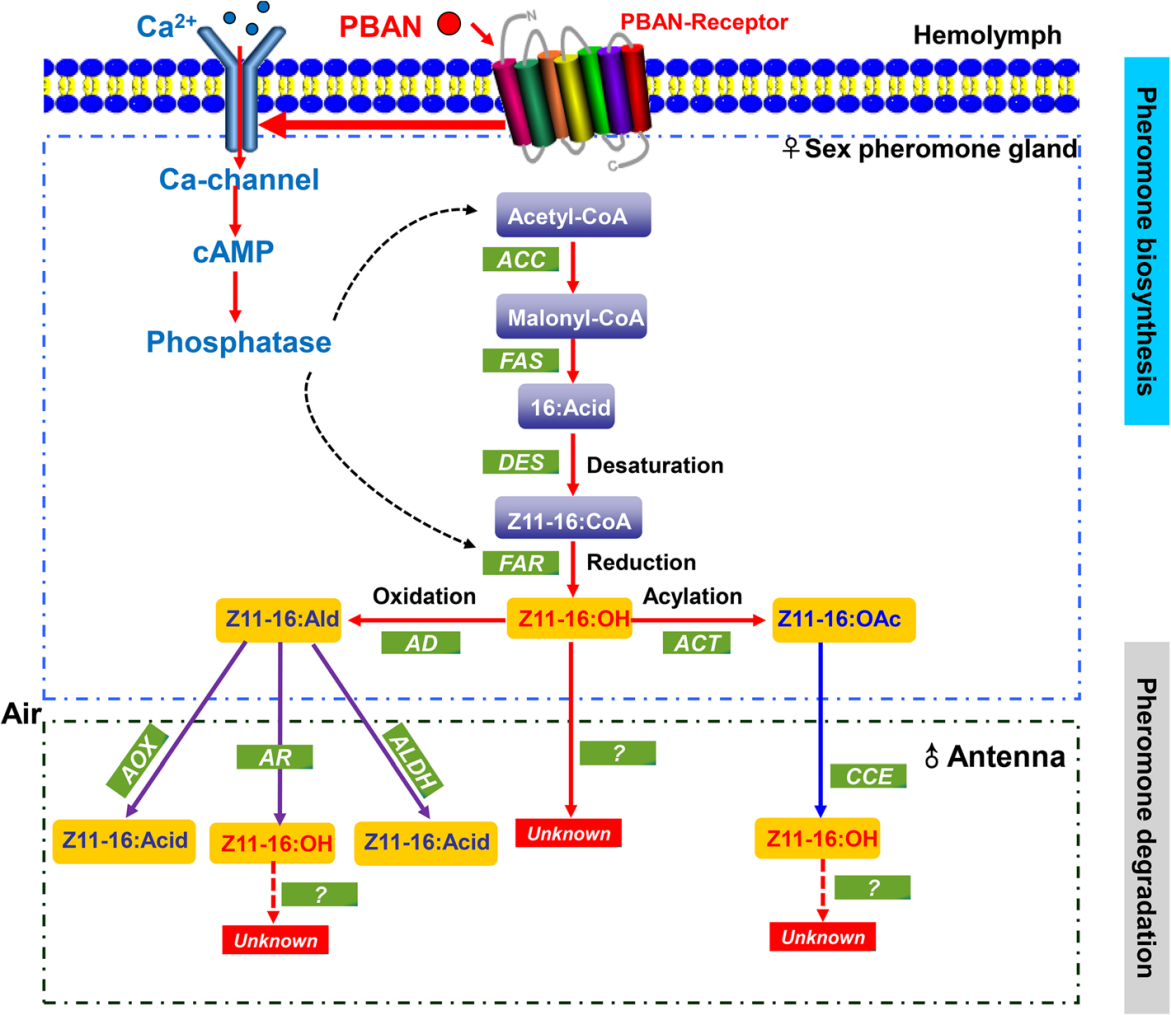
Proposed biosynthetic pathway of sex pheromone production in female DBM moths (adapted from [2, 16, 77])

The putative pathways of pheromone biosynthesis and degradation are shown in Fig. 2. We identified a number of genes related to the pathways by using the BLASTx search. Below, we describe the specific tissue expression patterns of these genes using qPCR.

### Genes involved in the sex pheromone biosynthesis pathway

#### Pheromone biosynthesis activating neuropeptide receptor (PBANr)

**Fig. 3:**
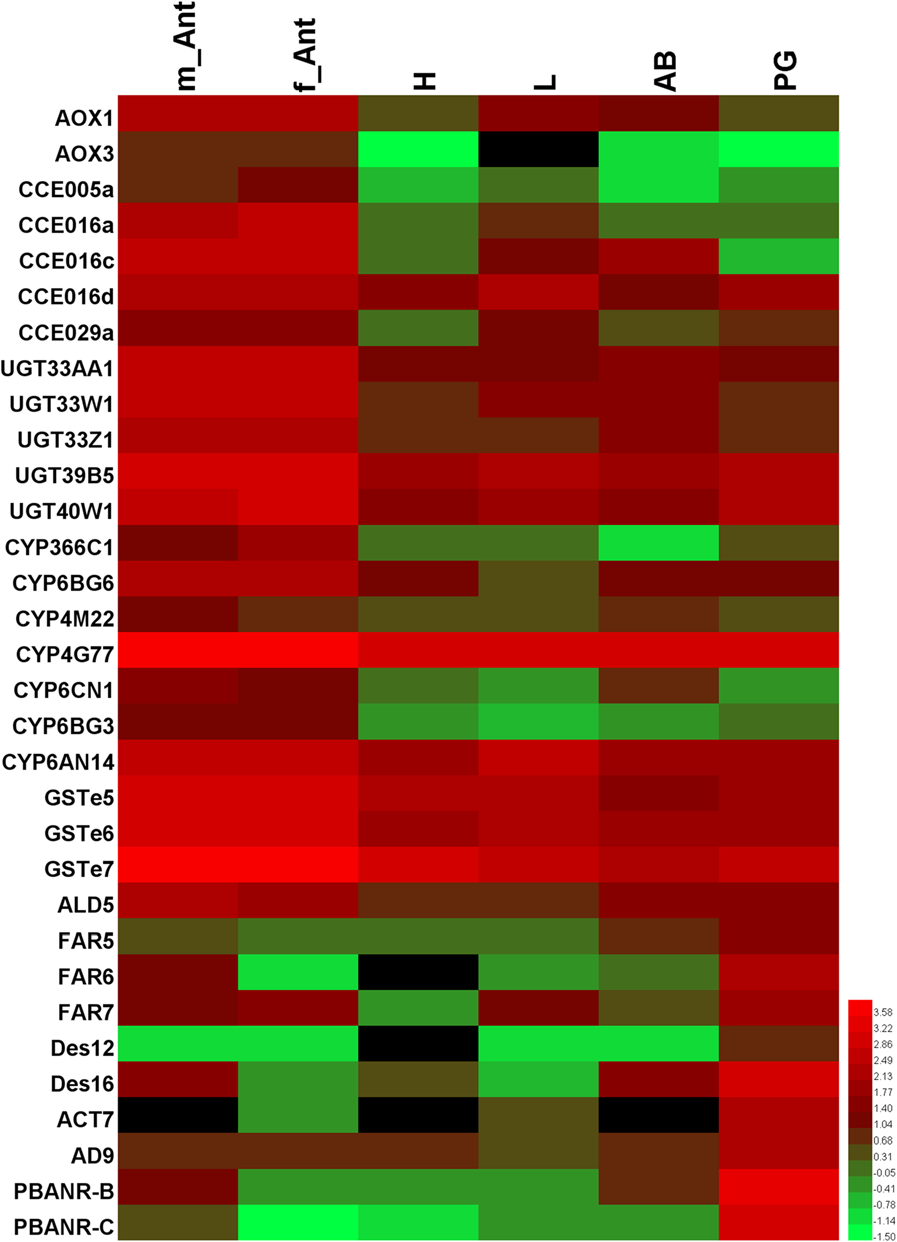
Heat map illustration of the tissue expression profile of sex pheromone biosynthesis and degradation genes based on FPKM values. m_Ant, male antennae; f_Ant, female antennae; H, Head; L, Leg; AB, Abdomen; PG, female pheromone gland. Gene names are indicated on the left. Scale Bar on bottom right indicates the degree of expression

Insect pheromone biosynthesis is regulated by PBANr that binds to PBAN released from the suboesophagal ganglion in the brain [5]. To date, no more than four alternative splice transcripts such as isoforms As, A, B and C with differences in their C-terminal sequences have been reported in one insect species [57]. B and C typically consist of the YXXΦ endosomal sorting motif and the complete seven transmembrane regions [58]. Although all four PBANrs are expressed dominantly in PGs, only PBANR-B and PBANR-C have been identified as intracellularly localized with some cell surface localization. PBANR-As and PBANR-A on the other hand, were completely intracellular, suggesting that PBANR-B and PBANR-C could be principal receptors participating in the PBAN signal reception [57]. In this study, we found two additional alternative splice transcripts (Px002138 and c63176_g2; see Additional file 3: Table S2) that possessed C-termini different from DBM PBANr genes previously described in the genome [59, 60] (Additional file 4: Figure S2). We named them as PluxyPBANR-C, −B and -A. Multiple amino acid sequence alignment of these PBANr isoforms showed the presence of typical PBANr sequence features (Additional file 4: Figure S2). We further analyzed the tissue expression pattern of PluxyPBANR-C and -B by comparing their FPKM values (Additional file 3: Table S2 and Fig. 3) in the transcriptomes from the different tissues. The results showed at least 3- and 5-fold higher numbers for PluxyPBANR-C and -B transcripts, respectively in PG than in other tissues studied, and is consistent with reports from other moths such as *Agrotis ipsilon* [61], *B. mori* [62] and *Heliothis virescens* [63].

#### Acetyl CoA Carboxylase (ACC) [EC:6.4.1.-]

The first step in moth pheromone biosynthesis is the ATP-dependent carboxylation of acetyl-CoA to malonyl-CoA by ACC [4]. This is a rate-limiting step in fatty acid biosynthesis [51]. In our transcriptome data set, seven ACC transcripts were identified (Additional file 3: Table S2) with all showing robust expression in the abdomen followed by PG (Additional file 3: Table S2). Among these, *PxACC3*, *PxACC4* and *PxACC5* showed higher expression than the others in PGs. In addition, homology analysis revealed that PxACC3 shared 73% amino acid identity with the ACC homolog from *Papilio xuthus* (Genbank XP_013176189.1), 70% with *Helicoverpa assulta* (Genbank (AKD01722.1) and 66% with *D. plexippus* (Genbank EHJ72299.1). *PxACC4* is a full-length ACC, and the predicted protein from this transcript shared a high amino acid identity (83%) with the ACC from *Agrotis segetum* (AID66639.1) [64]. Similar to *A. segetum* ACC, *PxACC4* also had dominant expression in the abdomen as well as in PG but without significant difference. PxACC5 also shared a high amino acid sequence identity (83%) with ACC from *B. mori* (XP_004930758.1) indicating a similar function for ACC homologs.

#### Fatty acid synthase (FAS) [EC:2.3.1.-]

In moths, saturated fatty acid is produced from malonyl-CoA and NADPH in a reaction catalyzed by FAS [4]. In this study, we found seven putative transcripts with high amino acid identities to known FAS genes (*PxFAS1-PxFAS8*) (Additional file 3: Table S2). Only one partial FAS transcript was found in the genome annotation, two novel transcripts were identified via genome mapping and four new FAS transcripts were found among the unigenes after *de novo* assembly. None of the seven *PxFAS* genes showed dominant expression in PGs (Additional file 3: Table S2). Five of the PxFASs displayed abdomen biased expression, unlike the two homologs, FAS from *A. ipsilon* (AGR49310.1) and *A. segetum* (AID66645.1) that shared 71 and 72% amino acid identity with PxFAS5 and PxFAS1, respectively and were expressed higher in PGs than in the abdomen [61, 64].

#### Fatty Acid Transport Protein (FATP) [EC:6.2.1.-]

The fatty acid transport protein (FATP) has been functionally characterized by in vitro expression and knock down assay in *B. mori* [65] and *Eilema japonica* [66]. These studies showed that FATP catalyzes the ATP-dependent esterification of extracellular long-chain fatty acids in sex PGs to produce the corresponding acyl-CoA derivatives. Four *PxFATPs* unigenes were found in this study; PxFATP1 shared high amino acid identity (74%) with FATP from *E. japonica* (BAJ33523.1); PxFATP2 and 4 shared 58 and 81% amino acid identity, respectively with FATP2 and FATP3 from *Sesamia inferens* [36]. All three *PxFATPs* displayed ubiquitous expression (Additional file 3: Table S2) indicating the lack of specialized function in the sex pheromone biosynthesis pathway. *PxFATP3* was not detected in any tissue in our transcriptome data, and thus maybe a pseudogene (Additional file 3: Table S2).

#### Desaturase (DES) [EC:1.14.19.-]

**Fig. 4:**
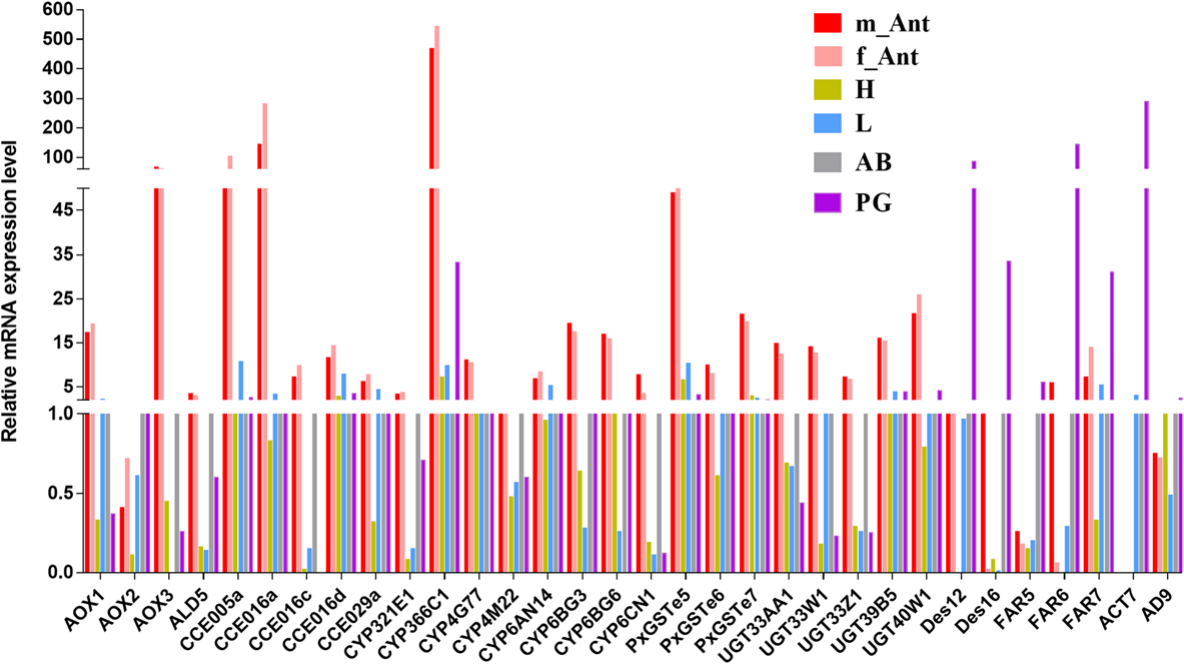
Relative expression levels of sex pheromone biosynthesis and degradation gene transcripts with antennae or pheromone gland-biased expression in different adult tissues. m_Ant, male antennae; f_Ant, female antennae; H, Head; L, Leg; AB, Abdomen; PG, female pheromone gland

**Fig. 5:**
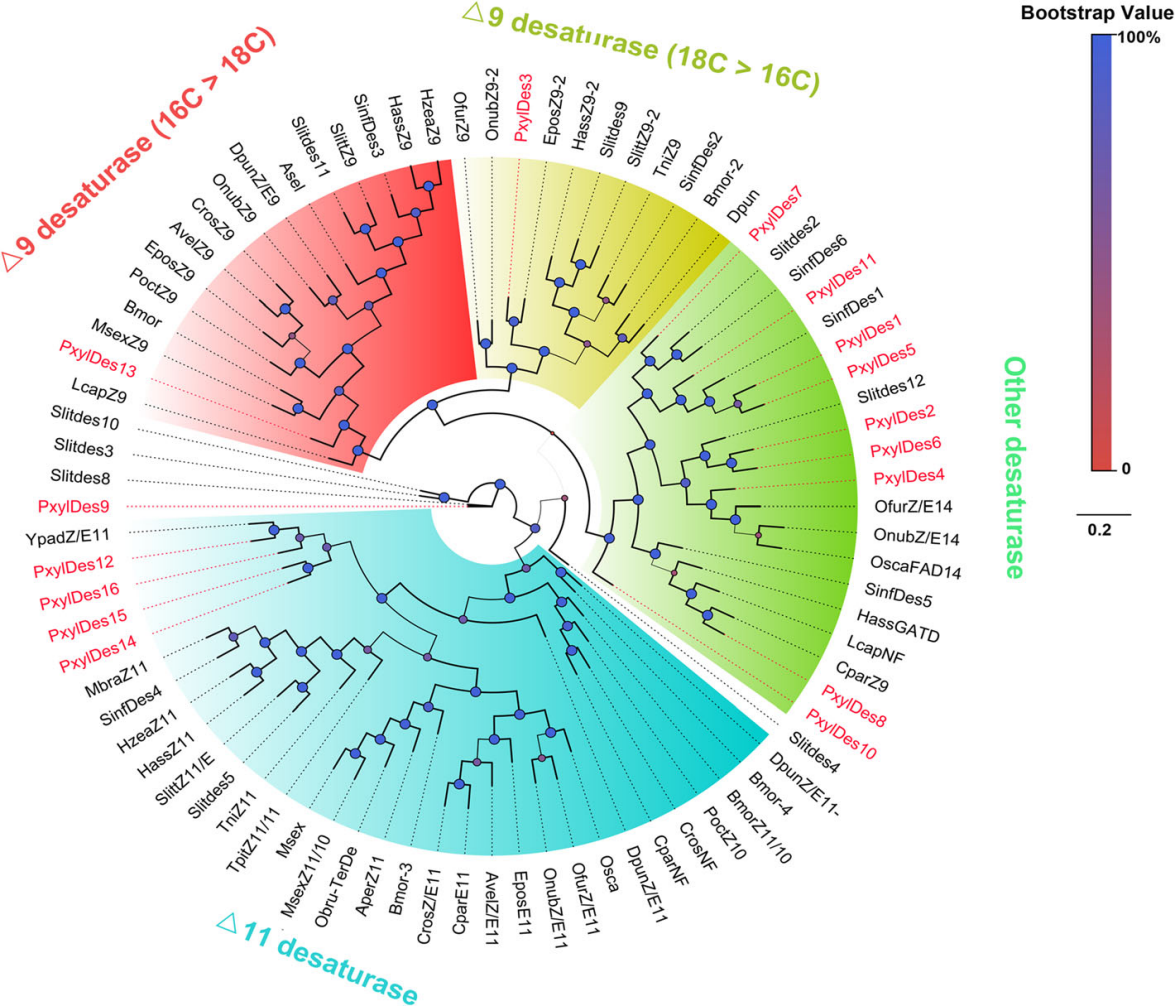
Phylogenetic tree of typical insect DES genes. PxylDES are highlighted in *red*. Species abbreviations: Bmor. *B. mori*; Slit. *Spodoptera litura*; Sinf. *Sesamia inferens*; Tni. *T. ni*; Ofur. *O. furnacalis*; Onub. *O. nubilalis*; Osca. *O. scapulalis*; Hass. *H. assulta*; Lcap. *L. capitella*; Cpar. *C. parallela*; Dpun. *D. punctatus*; Cros. *C. rosaceana*; Obru. *O. brumata*; Aper. *A. pernyi*; Msex. *M. sexta*; Tpit. *T. pityocampa*; Hzea. *H. zea*; Mbra. *M. brassicae*; Ypad. *Y. padellus*; Poct. *P. octo*; Epos. *E. postvittana*; Avel. *A. velutinana*; and Asel. *A. selenaria cretacea*

Typically moth pheromones contain double bonds, which require desaturases to introduce the bonds into specific locations in the fatty acyl carbon chains [15, 67, 68]. Depending on the locations of the double bonds introduced, DES can be classified into the following major groups: Δ5 [6], Δ6 [7], Δ9 [8–12], Δ10 [13, 14], Δ11 [9, 15, 16] and Δ14 [17, 18]. The major sex pheromone components in DBM are Z11-16:Ald, Z11-16:OAc and Z11-16:OH [48, 51, 52]. A single Z11-16:Ald can trigger copulation behaviour in male moths [48, 69]. Thus, Δ11 desaturase is expected to play a key role in this pheromone component biosynthesis. Investigation of the presence of desaturases in our transcriptome data set using genome mapping and *de novo* transcriptome assembly revealed 16 *PxylDes* transcripts (Additional file 3: Table S2). FPKM and qPCR analysis revealed that* PxylDes12* and *16* were expressed abundantly in PG (Additional file 3: Table S2, Figs. 3 and 4).﻿ In addition, phylogenetic analysis showed that PxylDes12, 14, 15 and 16 clustered into the Δ11 clade, which also had the *SinfDes4* homolog from *S. inferens* [36] (Fig. 5). Thus, we propose that the two PxDes may be involved in the desaturation of Z11-16:CoA, the major sex pheromone component in DBM. Ten additional PxylDes were assigned to other desaturase groups and also did not have PG biased expression. These genes may also be involved in other physiological functions rather than sex pheromone component desaturation.

#### Fatty Acyl Reductase (FAR) [EC:1.2.1.-]

**Fig. 6:**
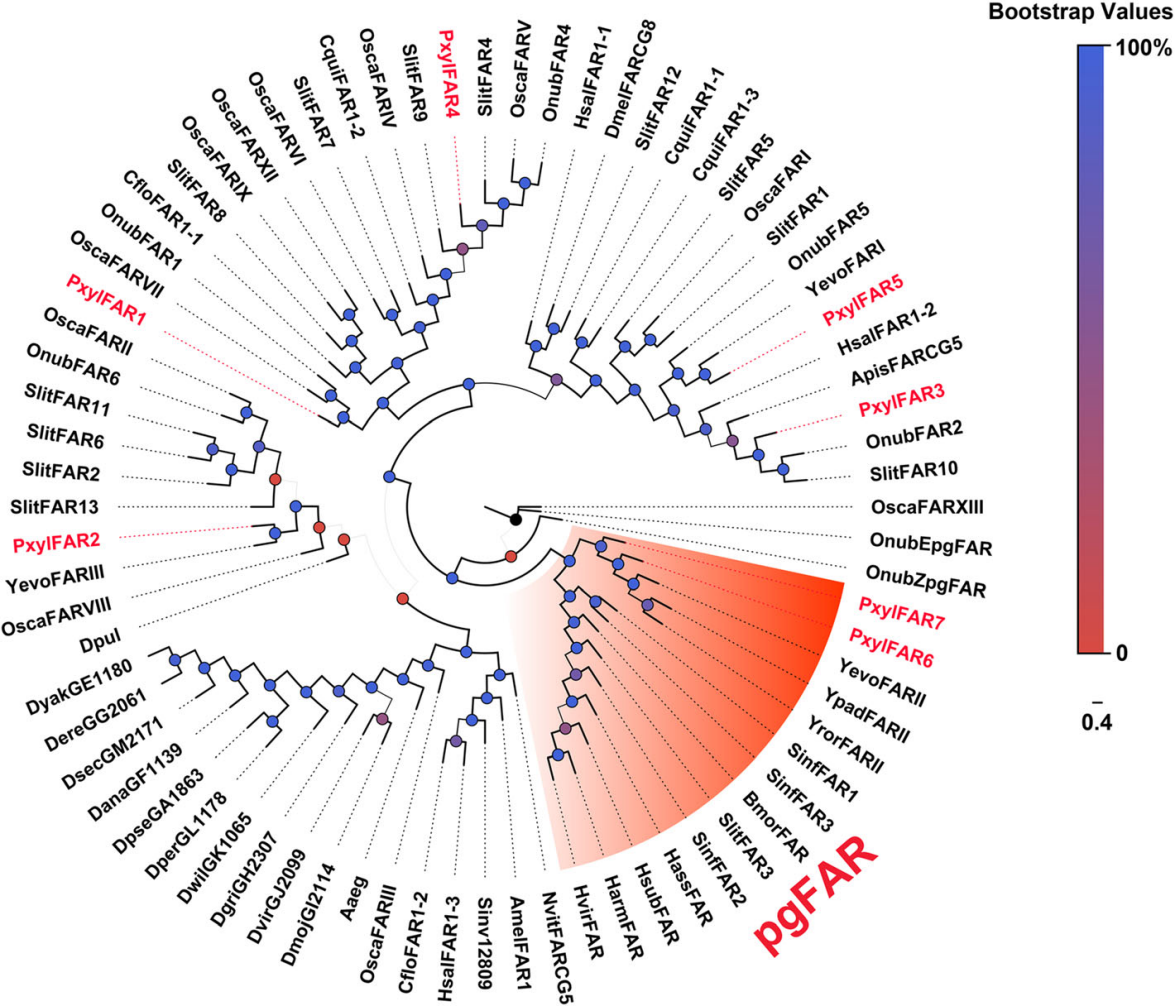
Phylogenetic tree of typical insect FAR genes. PxFARs are highlighted in *red*. Species abbreviations: Bmor. *B. mori*; Cqui. *C.ulex quinquefasciatus*; Yevo. *Y. evonymellus*; Ypad. *Y. padellus*; Yror. *Y. rorrellus*; Hass. *H. assulta*; Harm. *H. armigera*; Hsub. *H. subflexa*; Hvir. *H. virescens*; Nvit. *N. vitripennis*; Amel. *A. mellifera*; Sinv. *S. invicta*; Hsal. *H. saltator*; Cflo. *C. floridanus*; Osca. *O. scapulalis*; Aaeg. *Aedes aegypti*; Dmoj. *D. mojavensis*; Dvir. *D. virilis*; Dgri. *D. grimshawi*; Dwii. *D. willistoni*; Dper. *D. persimilis*; Dpse. *D. pseudoobscura*; Dana. *D. ananassae*; Dsec. *D. sechellia*; Dere. *D. erecta*; Dyak. *D. yakuba*; Dpul. *D. pulex*; Dmel. *D. melanogaster* and Apis. *A. pisum*

In DBM, Z11-16: OH is a main sex pheromone component [51, 52], which is produced by the reduction of fatty acyl precursor, Z11-16:CoA, in an NADPH dependent reaction [22, 70–72]. In this study, we identified in total, seven putative FAR transcripts, named *PxFAR1*-*7* (Additional file 3: Table S2). Among them, *PxFAR6* and *PxFAR7*, clustered with the pgFAR clade (Fig. 6). All FARs in this group displayed PG dominant expression, including the first identified *pgFAR* from *B. mori* [70], *SlitFAR3* from *S. litura* [73] and *SinfFAR2* from *S. inferens* [36]. Together, the FPKM value and qPCR analysis showed that among the seven *PxFARs, PxFAR6* had the highest expression (20-fold higher) in PGs when compared to the other tested tissues (Figs. 3 and 5). Therefore, *PxFAR6* may likely play a major role in the conversion of Z11-16:CoA to Z11-16:OH in DBM. In addition, we found another FAR predominantly expressed in PG; *PxFAR5* that did not cluster with the pgFAR group. It shared high amino acid identity (71%) with *YevoFARI* from *Yponomeuta evonymellus* (ADD62438.1). A previous report on the PG specific expression of *YevoFARI* determined by qPCR analysis was also not clear [22]. The expression and functional analyses of this clade need further investigation.

#### Acetyltransferase (ACT)

ACT is known to convert fatty acid alcohol to the corresponding ester in plants [74–76]. However, no ACT related to sex pheromone component biosynthesis has been thus far identified at the molecular level. In this study, we identified 21 ACT transcripts, *PxylACT1-22*, by homolog searches using sequences (BLASTp) obtained from our previous studies as query; SlitACTs from *S. litura* [73], SinfACTs from *S. inferens* [36] and AsegACTs from *A. segetum* [64] (Additional file 3: Table S2). We found that only *PxACT7* displayed PG abundant expression via FPKM and qPCR analysis (Additional file 3: Table S2, Figs. 3 and 4). Further, BLASTx showed that PxACT7 shared a low amino acid identity (39%) with AsegACT (AIN34700.1) [64], which was shown to lack esterification activity by using in vitro yeast expression system and GC-MS analysis [65]. Not surprisingly, AsegACT was not abundant in PG, and its FPKM value in PG was almost equal to that in the abdomen [15]. Three ACTs from *Agrotis ipsilon*, *ATF-KC007357*, *ATF-KC007358* and *ATF-KC007360*, were mainly expressed in PG than the rest of the body [61]. Their homologs in DBM are PxACT17, 19 and 14, which shared 65, 75 and 91% amino acid identities, respectively. However, none of these displayed PG dominant expression (Additional file 3: Table S2).

#### Alcohol dehydrogenase (AD)

Alcohol dehydrogenase converts alcohol compounds to corresponding aldehydes. However, no alcohol dehydrogenase has been identified at the molecular level from insect PGs although biochemical studies have shown the presence of alcohol dehydrogenses in the PGs of several species such as *Heliothis virescens* [77], *Helicoverpa armigera* [78] and *H. assulta* [78]. Z11-16:Ald was found to be a major sex pheromone in DBM, indicating that AD plays a crucial role in its biosynthesis. In this study, we conducted a BLASTp analysis by using previously identified SinfADs from *S. inferens* [36], AipsADs from *A. ipsilon* [61], CpomADs from *Cydia pomonella* [79]*,* HassADs from *H. assulta* [78], HarmADs from *H. armigera* [78] and HvirAD from *H. virescens* [77]. We found in total, 22 ADs in DBM, named *PxylAD1-22* (Additional file S3: Table S2). The FPKM values of these 22 *PxylADs* revealed that only one AD (*PxylAD9*) had PG dominant expression. qPCR assay also confirmed this (Figs. 3 and 4) and showed at least 2-fold higher expression in PG than in other tested tissues (Fig. 4). We also found several homologs of previously identified ADs from *H. armigera* (*HarmAD5*) and *H. assulta* (*HassAD6*) that had PG biased expression [78]. In our study, PxylAD15 shared the highest amino acid identity 70.2% with HassAD6, but PxylAD15 had not PG predominant expression. But, PG-enriched PxylAD9 shared only 25.1% amino acid identity with HassAD6.

### Sex pheromone and other odorant-degrading enzyme genes

#### Aldehyde oxidase (AOX)

**Fig. 7:**
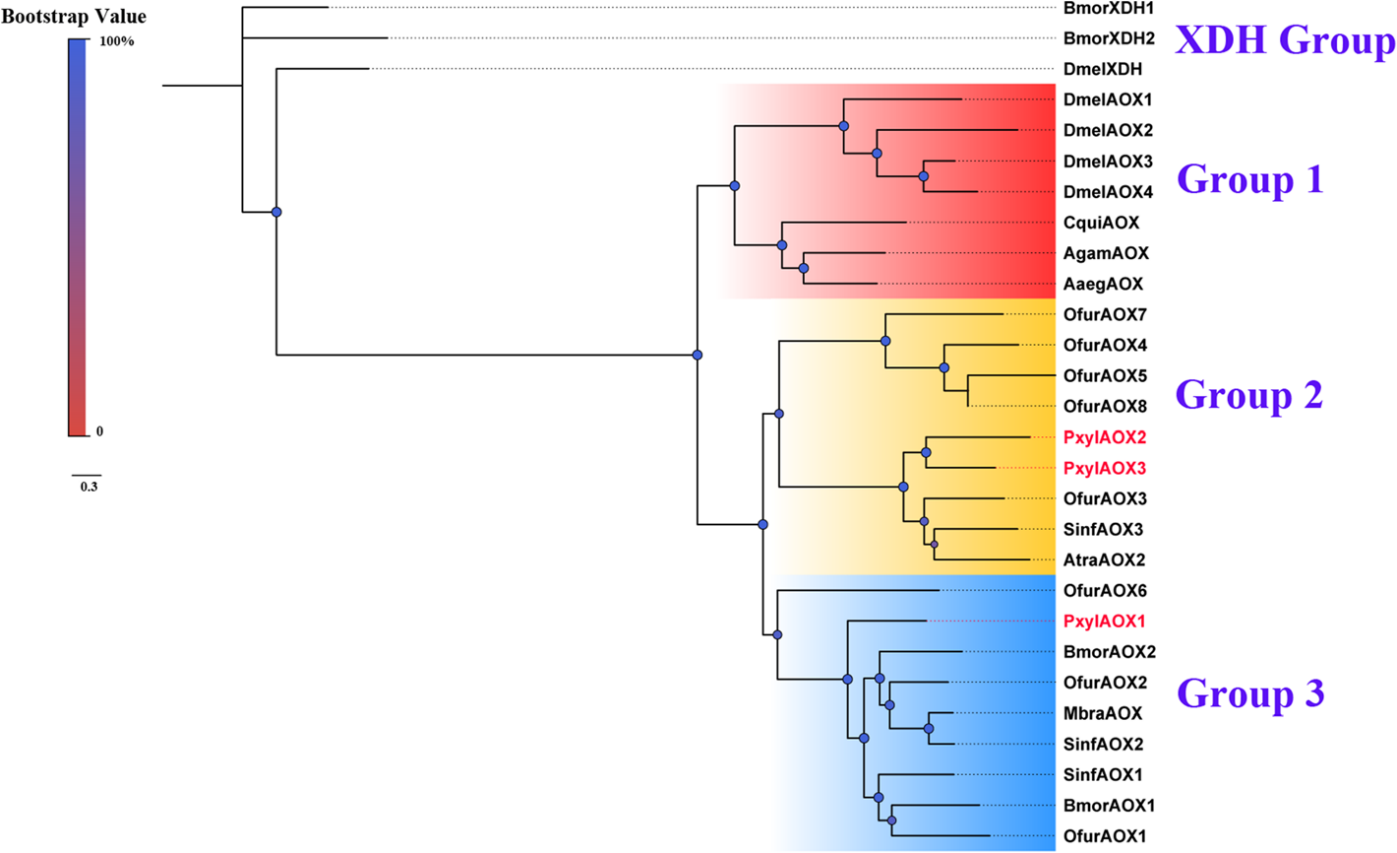
Phylogenetic tree of typical insect AOX genes. PxylAOXs are highlighted in *red*. Species abbreviations: Dmel, *D. melanogaster*; Bmor, *B. mori*; Cqui, *C quinquefasciatus*; Agam, *A. gambiae*; Aaeg, *A. aegypti*; Ofur, *Ostrinia. furnacalis*; Sinf, *S. inferens*; Atra, *A. transitella* and Mbra. *Mamestra. brassicae*. Scale bar on *top left* indicates the color code for bootstrap values in the phylogenetic tree

Aldehyde oxidase is considered as a sensillum enzyme that degrades redundant aldehyde odorants (including aldehyde sex pheromone components) to corresponding acids without the need for coenzymes [31–33]. We found three full-length AOXs in the DBM transcriptomes via genome mapping. These were named *PxylAOX1*, *PxylAOX2* and *PxylAOX3* (Additional file 3: Table S2), all containing the typical conserved motifs: a FAD-binding domain, two putative iron-sulfur (2Fe-2S) redox centers, and a molybdenum-cofactor (MoCo)-binding site. One 2Fe-2S redox center contains four conserved cysteine residues that coordinate with iron. *PxylAOX1* and *PxylAOX3* likely originated from a gene duplication event because they were located in the same scaffold (scaffold_267). Based on FPKM values and qPCR analysis, *PxylAOX1* and *PxylAOX3* were identified to have antennae enriched expression without sex-bias; *PxylAOX1* expression in the antennae was more than 60-fold higher and *PxylAOX3* expression was 40-fold higher than in other tissues*.* In addition, *PxylAOX1* expression was higher than *PxylAOX3* (Additional file 3: Table S2, Figs. 3 and 4), while *PxylAOX2* showed ubiquitous expression and therefore may perform a more generalized function. We also noticed a putative AOX identified in the DBM genome (ID: CCG014884.1) [54]. This sequence was identified as a xanthine dehydrogenase (XDH) member by BLASTx search. A phylogenetic tree constructed by using known insect AOXs and XDH as an out group (Fig. 7) showed the distribution of insect AOXs into three major groups; group 1 consisted of dipteran AOX, group 2 had PxylAOX2 and PxylAOX3, and group 3 had AtraAOX2 from *A. transitella* and their homologs, which were shown to degrade the aldehyde sex pheromone component, Z11Z13–16Ald and several aldehyde plant volatiles [33], and *SinfAOX2* from *S. inferens* with antennae dominant expression [36]. PxylAOX1 clustered with group 3 along with *BmorAOX1* [80], *BmorAOX2* [80], *SinfAOX1* [36], *SinfAOX2* [81], and *MbraAOX* [31], all of which have antennae enriched expression pattern. However, like other AOXs, *PxylAOX1* and *3* did not show sex biased expression suggesting that they may be specifically involved in aldehyde plant volatiles degradation rather than sex pheromone component pathways.

#### Aldehyde dehydrogenase (ALDH) and Aldehyde reductase (AR)

Unlike AOX, both ALDH and AR are thought to be intracellular and NAD(P)^−^ dependent enzymes that catalyze the oxidation and reduction of a broad spectrum of aldehyde substrates to their corresponding acids and alcohols, respectively. Five *PxylALDHs* (named *PxylALDH1-5*) were identified from the DBM transcriptome data set. Among them, only *PxylALDH4* was mainly expressed in the antennae as determined by the FPKM value and qPCR analysis (Additional file 3: Table S2). The expression level of *PxylALDH4* in PG was at least 2-fold higher than in other tested tissues, indicating its likely function in odorant-degradation. Similar to AOX, this gene also showed no sex-biased expression suggesting that it is involved in plant volatiles degradation. A previous study showed that one ALDH, *AaALDH3,* oxidizes farnesal into farnesoic acid in *Aedes aegypti* [39]. But this gene only shared 11% amino acid identity with PxylALDH4, suggesting that they could act on different substrates. The ARs in general, include four reductase superfamilies; medium-chain dehydrogenases/reductases (MDR), short chain dehydrogenases/reductases (SDR), aldo-keto reductases (AKR) and NADB (NAD^+^ binding) reductases. Through motif and BLAST analyses, we found in total, 15 ARs among which were six MDRs (*PxylAR2*, *3*, *5*, *6*, *14* and *15*), eight AKRs (*1, 7, 8, 9, 10, 11, 12* and *13*) and one NADB (*PxylAD4*). However, no SDR was found (Additional file 3: Table S2). In *B. mori* [82] and *H. armigera* [38], two AKRs have been characterized in vitro; these were reductases that converted the sex pheromone components, E10,Z12-16:Ald (*B. mori*), Z9-16:Ald (*H. armigera*) and Z11-16:Ald (*H. armigera*) to corresponding alcohols. FPKM values did not show AR expression specific to the antennae. Two AKRs shared the most amino acid identities with PxylARs; these were PxylAD9 (59%) and PxylAD7 (37%), but *PxylAD9* and *PxylAD7* displayed ubiquitous expression, indicating their involvement in other physiological functions instead of olfaction alone.

#### Carboxyl/cholinesterase (CCE)

**Fig. 8:**
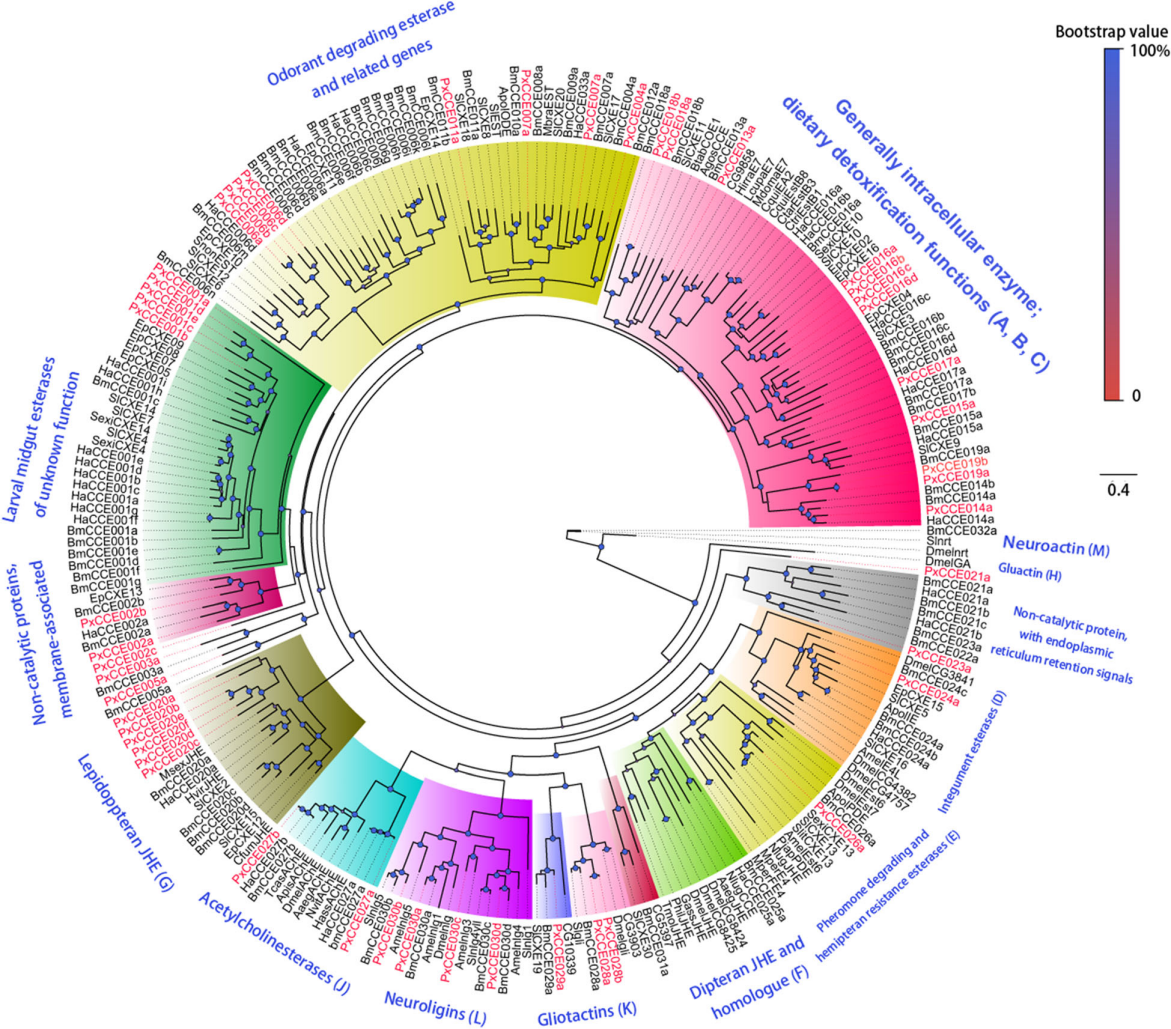
Phylogenetic tree of typical insect CCE genes. PxCCEs are highlighted in *red*. Species abbreviations: Dmel. *D. melanogaster*; Bmor. *B. mori*; Agos. *Aphis gossypii*; Hirr. *Haematobia irritans*; Lcup. *Lucilia cuprina*; Mdom. *Musca domestica*; Cqui. *Culex quinquefasciatus*; Ctar. *Culex tarsalis*; Ctri. *Culex tritaeniorhynchus*; Ha. *H. armigera*; Sexi. *Spodoptera exigua*; Sl. *S. littoralis*; Slit. *Spodoptera litura*; Ep. *Epiphyas postvittana*; Apol. *Antheraea polyphemus*; Amel. *Apis mellifera*; Pjap. *Popillia japonica*; Nlug. *Nilaparvata lugens*; Mper. *Myzus persicae*; Aaeg. *Aedes aegypti*; Gass. *Gryllus assimilis*; Phil. *Psacothea hilaris*; Tmol. *Tenebrio molitor*; Apis. *Acyrthosiphon pisum*; Tcas. *Tribolium castaneum*; Hass. *Heliothis assulta*; Nvit. *Nasonia vitripennis*; Cfum. *Choristoneura fumiferana*; Hvir. *Heliothis virescens*; Mbra. *M. brassicae* and Msex. *Manduca sexta*

**Fig. 9:**
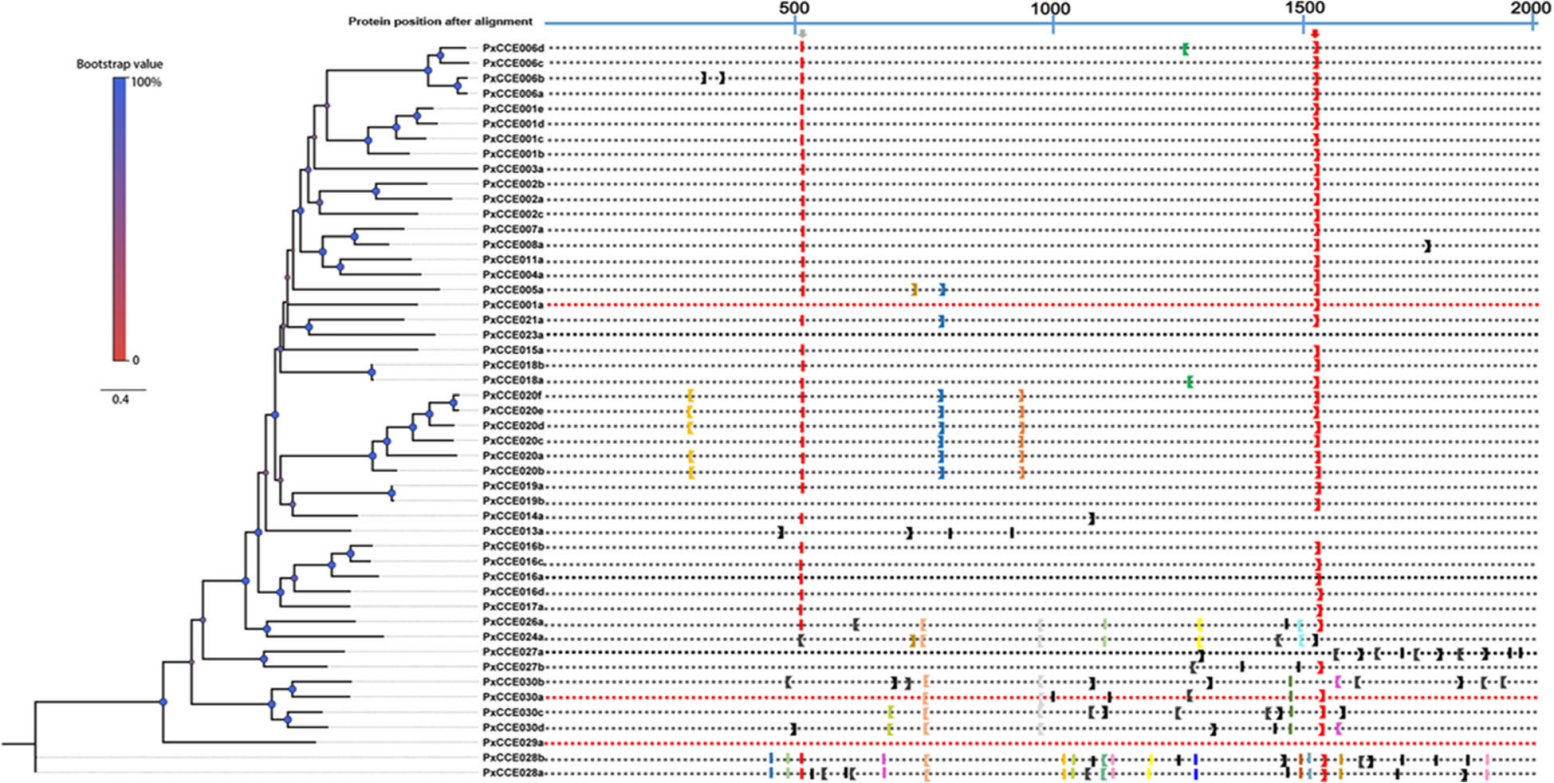
Phylogenetic tree and intron positions of PxCCE genes. The nomenclatures of clades are based on previously published data. The intron positions in sequences are shown as (|) for a phase 0 intron, ([) for a phase 1 intron and (]) for a phase 2 intron. *Arrows* indicate the most conserved intron insertion site in PxCCEs. Same *color brackets* indicate the same intron insertion site among different genes

Insect CCE is a superfamily, which participates in many physiological processes and contains various substrates [83]. Some of them are secretory enzymes that refresh ORs by clearing redundant esterase odorants around them. Besides Z11-16:Ald, Z11-16:Ac is another major sex pheromone component reported in *P. xylostella* [48]. We found 49 CCEs in the DBM transcriptome that was mapped to the reference genome (Additional file 3: Table S2). This total number is lower than 69 identified in *B. mori* [84, 85], but higher than 39 found in *H. armigera* [86]. A phylogenetic tree of known insect CCEs is shown in Fig. 8. These CCEs were named from CCE001-30 sub-groups based on the *B. mori* [84, 85] and *H. armigera* [86] 13 major clade nomenclature system. By FPKM values and qPCR analysis, five CCEs were found to have antennae enriched expression (Additional file 3: Table S2, Figs. 3 and 4); PxCCE005a, 016a, 016c, 016d and 029a. These were distributed into three clades. PxCCE016a, 016c and 016d were located in the same sub-clade containing most general intracellular enzymes with dietary detoxification functions or ester odorant degradation functions such as the two CCEs with antennae dominant expression, SlCXE10 [87] and SexiCXE10 [40], that degrade ester plant volatiles. Unlike these two CCEs, *PxCCE016a*, *016c* and *016d* had no obvious sex biased expression. Other CCEs involved in non-olfactory functions have been described in some previous studies [88–90], We found the homologs of these genes among the *PxCCEs*, and they did not show antennae dominant expression, thus they may not participate in olfaction. For instance, in Group E, the pheromone degrading esterase, ApolPDE from *A. polyphemus* [91], PjapPDE from *Popillia japonica* [92], SexiCXE13/SlitCXE13 from *S. exigua* and *S. litura* [43], and DmelEst6 from *D. melanogaster* [93] were able to hydrolyze ester sex pheromone components. These genes showed abundant expression in the antennae except SexiCXE13 and SlitCXE13. PxCCE026a was their closest homolog, but it showed ubiquitous expression by FPKM analysis. Moreover, PxCCE016b also showed ubiquitous expression. A recent study showed that PxCCE016b was involved in chlorantraniliprole resistance; its increased expression in resistant strains enabled better degradation of ester pesticides [94]. The four 016 sub-group CCEs are derived from the same ancestor because they clustered in the same clade in the phylogenetic tree and may have evolved to perform various physiological functions based on the difference in tissue expression patterns between *PxCCE016b* and three other *PxCCE016* CCEs. PxCCE029a and PxCCE005a were in the uncharacterized functional clade. The 029 clade CCEs lacked one or more of the enzyme catalytic active site residues, but had another active site called carboxyl/cholinesterase-like adhesion molecules including transmembrane and binding domains [95]. Thus, PxCCE029a may be involved in neuro/developmental functions. We also investigated the intron-exon organization in *PxCCEs* (Fig. 9)*.* The average intron size was 1290 nt, which was slightly lower than 1372 in *B. mori* [85]. The longest intron was between exons 2 and 3 in *PxCCE027b* and comprised 10,014 nt, similar to that observed in *BmCCE027b* [85]. The shortest intron was in *PxCCE028b* between exons 8 and 9, and contained only 33 nt. Further, the approximate distribution of introns is shown in Fig. 9, which shows the positions of introns in PxCCEs by multiple sequence alignment. We also collected and marked the splice sites into three phases according to their positions in the codons. We noticed the presence of two highly conserved intron insertion sites and the splice site phase was also conserved. The two most conserved introns were a phase 2 intron around position 1500 present in 42 CCEs (Fig. 9, red arrow) and a phase 0 intron around position 500 in 37 CCEs (Fig. 9, gray arrow). The two conserved intron insertion sites and splice site phases were identical to that reported in *B. mori* CCEs [85], indicating that these sites were likely conserved since the early stage of CCE evolution. In addition, several clade specific conserved sites were also observed and were similar to the clade-specific conservation of intron phase and position reported in *P. xylostella* GSTs [56]. All CCEs in clade 020 possessed two conserved introns, phase 2 introns at positions 700 and 800 (Fig. 9, blue and dark brown brackets, respectively). Notably, two intron insertions and phase splice sites were conserved in two distant clades, 024–026 and 030 (Fig. 9, light brown and gray brackets, respectively). These conserved introns were also observed in clades 020, 024, 026 and 030 in *B. mori* and the neuroligins of *D. melanogaster* and *A. mellifera* [85].

#### UDP-glycosyltransferases (UGT)

**Fig. 10:**
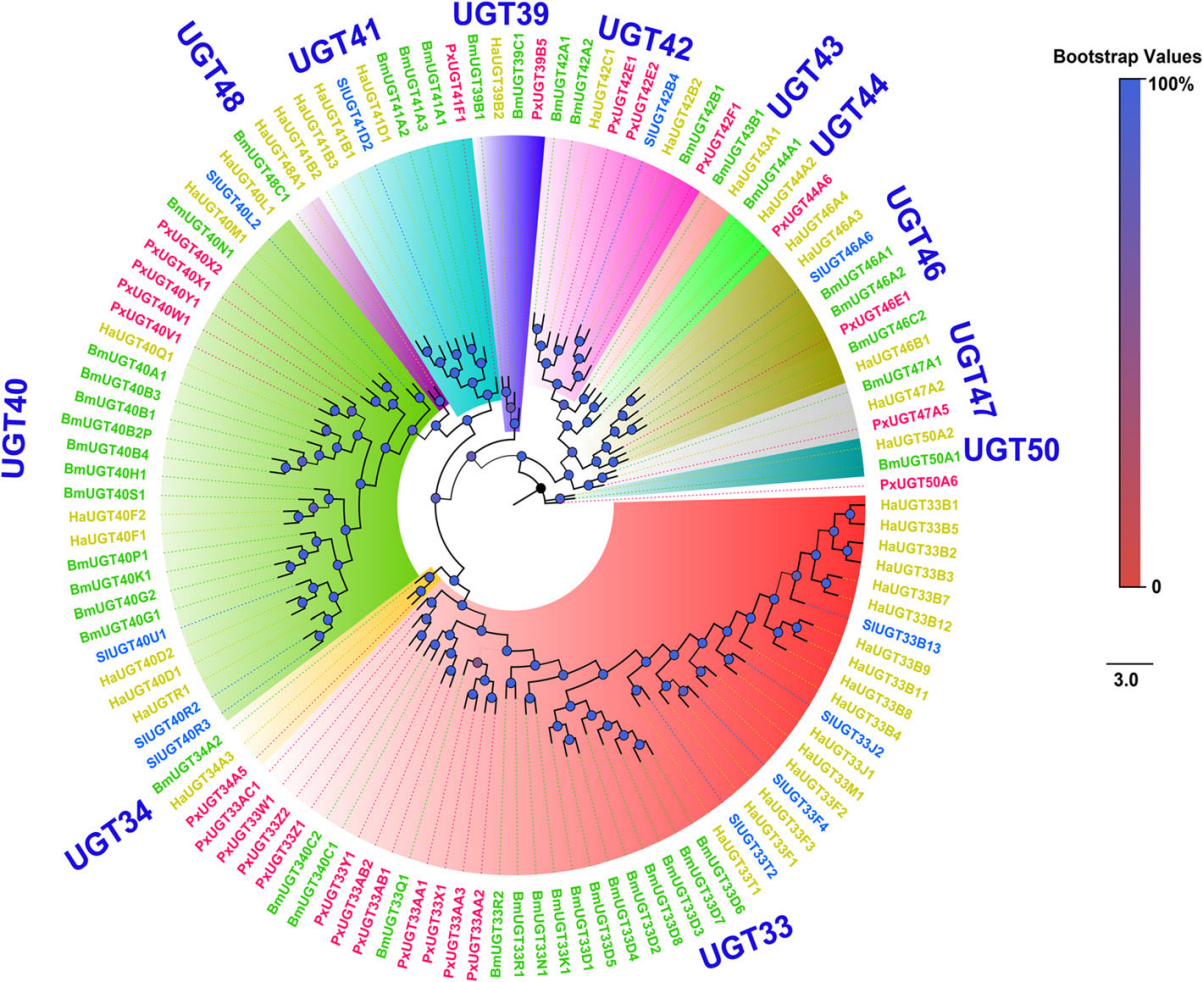
Phylogenetic tree of typical insect UGT genes. PxUGT*s* are highlighted in *red*. Species abbreviations: Bm. *B. mori*; Sl. *S. littoralis*; Ha. *H. armigera*

UGT is a superfamily of detoxifying enzymes that improve the excretion of certain molecules by transferring glycosyl residues from nucleotide sugars; for example, hydrophobic molecules are thus converted to hydrophilic molecules for easy excretion. Typical examples of such molecules in insects are odorant molecules [46, 96, 97]. In the present study, 26 UGTs were found via genome mapping and transcriptome assembly of all six transcriptomes from DBM (Additional file 3: Table S2). All PxUGTs presented the classic UGT signature motif (N-terminal substrate binding site and N-glycosylation sites) similar to that described for other insect UGTs [46, 97, 98]. These UGT sequences have been submitted to the UGT Nomenclature Committee (http://prime.vetmed.wsu.edu/resources/udp-glucuronsyltransferase-homepage) for nomenclature assignments [97] (the sub-group names are shown in Additional file 3: Table S2). To further uncover the putative functions of *PxUGTs*, a phylogenetic tree was constructed by using UGTs from *S. littoralis* [46], *B. mori* [98, 99] and *H. armigera* [98] (Fig. 10). To date, there are 14 UGT families identified in lepidopterans [98]. PxUGTs were distributed within 11 sub-families; none were in UGT34, 47 and 48 sub-families. UGT33 and UGT40 sub-families had the most members in lepidopteran species [46, 98]. Similarly, most PxUGTs were clustered in UGT33 (n = 10) and UGT40 (n = 4) sub-families. In the UGT33 sub-family, *BmUGT33D8* [99] and one UGT from *M. sexta* (AI234470.1) [100] displayed antennae enriched expression. By using FPKM values and qPCR analysis, we also identified three UGT33 family members among *PxUGTs* (*PxUGT33AA1*, *PxUGT33W1* and *PxUGT33Z1*) that displayed antennae dominant expression (Additional file 3: Table S2, Figs. 3 and 5) indicating their olfactory function. Recently, two more UGTs from UGT40 and 46 sub-families, *SlUGT40R3* and *SlUGT46A6* from *S. littoralis*, showed abundant expression in the male antennae after exposure of moths to the sex pheromone component, Z9E11-14:Ac or an ester plant volatile, Z3-6:Ac. This suggested that the two UGTs may be odorant-degrading enzymes. We also found a UGT40 member in DBM, *PxUGT40W1*, which displayed the highest expression in antennae than in other tissues. However, there was no significant difference in the expression of *PxUGT40W1* between male and female antennae, thus indicating that it may be involved in the removal of plant volatiles rather than sex pheromone components. In addition, *PxUGT39B5* of the UGT39 sub-family also had an antennae enriched expression pattern indicating its olfactory function. However, the functions of its homologs have not been reported yet.

#### Cytochrome P450

Cytochrome P450 is a multi-functional enzyme in organisms, and is involved in detoxification, resistance, and odorant clearance [44, 101]. A previous study annotated 85 P450 genes (*PxCYPs*) in DBM but the tissue specific expression pattern was investigated only in the larval stages but not in adult tissues [55]. In the present study, the 85 *PxCYPs* were analyzed in various adult tissue transcriptomes and validated via qPCR (Additional file 3: Table S2, Figs. 3 and 4). We found that eight *PxCYP*s had predominant expression in the antennae of both sexes, and belonged to three CYP sub-families, CYP3: *PxCYP321E1, 366C1;* CYP4*: PxCYP 4G77, 4 M22,* and CYP6: *PxCYP6AN14, 6BG3, 6BG6* and *6CN1* (Additional file 3: Table S2, Figs. 3 and 4). One CYP3 member, CYP345E2, which is an antenna-specific CYP from the pine beetle, *D. ponderosae*, was shown to catalyze the oxidation of monoterpene volatiles from pine hosts [44]. In our study, we found 2 CYP345E2 homologs in DBM; *PxCYP321E1* and *PxCYP366C1*, both with higher expression in the antennae. In addition, *PxCYP366C1* had the highest expression in the antennae when compared to other candidate odorant-degrading CYPs. This gene was also reported as highly expressed in adult heads from different developmental stages [55], indicating that PxCYP366C1 plays a vital role in specific olfactory behaviors of DBM adults; an example of such behavior could be locating oviposition sites. Some members of the CYP4 sub-family were also identified to be olfactory related. CYP4AW1 in *P. diversa* was reported to be involved in pheromone inactivation by analyzing its antennae specificity and through electrophysiological studies [45]. In DBM, we identified 2 CYP4 genes; *PxCYP4G77* and *4 M22* both with robust expression in the antennae. A previous study reported the high expression of *PxCYP4M22* in an insecticide-resistance strain than in the susceptible strain [55]. Together with its tissue specific expression pattern, we propose that *PxCYP4M22* may participate in insecticide detoxification in the antennae of the insecticide-resistance strain. In the CYP6 sub-family, no member has been reported to have odorant clearance function. However, several genes have been reported to have olfactory organ specific expression; for example CYP6W1 [102] and CYP6A20 [103] in *D. melanogaster*, and *CYP6B48*, *CYP6B42* and *CYP6AE49* expressed in the antennae of both sexes and male proboscis in *S. littoralis* [104]. We found four CYP6 members in DBM (*PxCYP6AN14, 6BG3, 6BG6* and *6CN1*) that were abundant in the antennae. *PxCYP6BG3* and *6BG6* had high expression in the larvae than in adults [55], suggesting that the two CYP genes may be involved in odorant clearance in larvae.

#### Glutathione S-Transferase (GSTs)

The functions of GSTs in many insect physiological functions such as insecticide resistance [56] and detoxification [105, 106] have been well-documented. In this study, we identified in total, 25 *PxGSTs* from the transcriptomes of DBM. In comparison with a previous study [56] we identified three new GSTs, *PxGSTe6*, *PxGSTe7* and *PxGSTu3* in DBM (Additional file 3: Table S2). Since *PxGSTu3* was not detected in the transcriptomes of all tested tissues we presume that this may be a pseudogene. One olfactory-specific GST from *M. sexta* named, GSTmsolf1, was reported to degrade the plant volatile, trans-2-hexenal [47]. When we used the deduced amino acid sequence of this protein as query in a BLASTp search, we found that it belonged to the GST sub-family Delta. However, all three antennae enriched *PxGSTs* we identified in this study belonged to the GST sub-family Epsilon (Additional file 3: Table S2, Figs. 3 and 4). We identified three new GSTs; *PxGSTe6*, *PxGSTe7* and *PxGSTe5*. Among these, *PxGSTe5* had the highest expression and was consistent with a previous report that showed robust expression of *PxGSTe5* in adults [56]. This suggested that *PxGSTe5* could participate in odorant degradation in the adult stage.

## Conclusions

Based on massive Illumina sequencing, in this study we sequenced the transcriptomes from six tissues of the diamondback moth. Genome mapping and *de novo* assembly of our DBM transcriptome data provides a reference gene set for sex pheromone biosynthesis pathway and degradation genes in this notorious agricultural pest. Further analysis of their tissue specific expression pattern via digital gene expression and qPCR validation revealed the identification of the following genes in the pheromone biosynthesis and degradation pathways. A) In the sex pheromone biosynthesis gene category, we identified two PBAN receptor isoforms with PG biased expression, 7 ACCs, 8 FASs, 4 FATPs, 16 DESs, 7 FARs, 21 ACTs and 22 ADs. Two PG abundant *PxDESs*, *PxDES12* and *16,* could be involved in Δ11 desaturation. Three possible candidate pgFARs, *PxFAR5*, *6* and *7*, were also identified to have PG dominant expression and could participate in the reduction of Z11-16:acid to Z11-16:OH. B) In the odorant/sex pheromone-degrading gene category, we identified three AOXs, five ALDHs, 15 ARs, 49 CCEs, 27 UGTs, 85 P450s and 26 GSTs. Among these genes, two AOXs, one ALDH, five CCE, five UGTs, eight P450s and three GSTs displayed antennae dominant expression, and were proposed to be involved in sex pheromone components and plant volatiles degradation. Together, our data provides a strong reference for the genes involved in sex pheromone biosynthesis and degradation pathways, and will be a useful gene set for future gene function studies. This data set may also provide potential gene targets to design pest control strategies based on disrupting pheromone-based behaviors of insect pests.

## Methods

### Insect samples

The 5^th^ instar larvae of DBMs were purchased from an insect rearing facility in Henan province, China. The rearing conditions were 27 °C, 14 L : 10D photoperiod and 65% ± 5% relative humidity (RH). We collected approximately 300 antennae, 300 heads, 300 legs, and 100 abdomens from 3-day old adults of both sexes (male/female = 1:1), and 100 female sex pheromone glands for transcriptome sequencing. Three biological replicates of these samples were also collected for qPCR. Ovipositors that were adhered to the abdomens were removed as much as possible to avoid tissue contamination. The tissue samples were stored in liquid nitrogen at −80 °C until further use.

### cDNA library construction and Illumina sequencing

Total RNA was extracted using TRIzol reagent (Invitrogen Carlsbad, CA, USA) according to the manufacturer’s protocol. RNA degradation and contamination was monitored on 1% agarose gels. RNA purity was checked using the NanoPhotometer spectrophotometer (IMPLEN, CA, USA). RNA concentration was measured using Qubit RNA Assay Kit in Qubit 2.0 Fluorometer (Life Technologies, CA, USA). RNA integrity was assessed using the RNA Nano 6000 Assay Kit of the Agilent Bioanalyzer 2100 system (Agilent Technologies, CA, USA). The cDNA library construction and Illumina sequencing of the samples were performed by Novogene Bioinformatics Technology Co. Ltd, Beijing, China. A total amount of 3 μg RNA was used as input material for the RNA sample preparations. Sequencing libraries were generated using NEBNext Ultra RNA Library Prep Kit for Illumina (NEB, USA) following manufacturer’s recommendations and index codes were added to assign sequences to each sample. Briefly, mRNA was purified from total RNA using poly-T oligo-attached magnetic beads. Fragmentation was carried out using divalent cations under elevated temperature in the NEBNext First Strand Synthesis Reaction Buffer (5×). First strand cDNA was synthesized using random hexamer primer and M-MuLV Reverse Transcriptase (RNase H-). Second strand cDNA synthesis was subsequently performed using DNA polymerase I and RNase H. Remaining overhangs were converted into blunt ends via exonuclease/polymerase activities. After adenylation of 3′ ends of DNA fragments, NEBNext Adaptor with hairpin loop structure were ligated to prepare for hybridization. In order to select cDNA fragments that were 150–200 bp in length, the library fragments were purified with the AMPure XP system (Beckman Coulter, Beverly, USA). Thereafter, 3 μL USER Enzyme (NEB, USA) was added to size-selected, adaptor-ligated cDNA and incubated at 37 °C for 15 min followed by 5 min at 95 °C. Then, PCR was performed with Phusion high-fidelity DNA polymerase, universal PCR primers, and index (X) primer. Finally, the PCR products were purified (AMPure XP system) and the library quality was assessed on an Agilent Bioanalyzer 2100 system.

### *De novo* assembly of short reads and gene annotation

Raw data (raw reads) in fastq format were first processed through in-house perl scripts. In this step, clean data (clean reads) were obtained by removing the adapter sequences, reads containing poly-N, and low quality reads from the raw data. Simultaneously, Q20, Q30, GC-content, and sequence duplication level of the clean data were calculated. All downstream analyses were based on clean data with high quality. *De novo* transcriptome assembly was performed using the short reads assembly program Trinity (version: r20140413p1) with min_kmer_cov set to 2 by default and all other parameters were set to default values. The overlap settings used for the assembly were 30 bp and 80% similarity, and all the other parameters were set to their default values.

Unigenes >150 bp were aligned by BLASTx with protein databases, including Nr, Swiss-Prot, KEGG, and COG (e-value < 10–5), to identify proteins with high sequence identity and to assign putative functional annotations. Next, we used the Blast2GO program (version: b2g4pipe_v2.5, e-value = 1.0E-6) (https://www.blast2go.com/) to obtain GO annotations of the unigenes and we obtained the GO functional classifications using the WEGO software (http://wego.genomics.org.cn/cgi-bin/wego/index.pl).

### Reads mapping to the reference genome

Reference genome and gene model annotation files were downloaded directly from the DBM genome website: http://iae.fafu.edu.cn/DBM/. Index of the reference genome was built using Bowtie v2.2.3 and paired-end clean reads were aligned to the reference genome using TopHat v2.0.12. We selected TopHat as the mapping tool to generate a database of splice junctions based on the gene model annotation file to yield a better mapping result compared to other non-splice mapping tools.

### Quantification of gene expression level

HTSeq v0.6.1 was used to count the read numbers mapped to each gene. Then, FPKM of each gene was calculated based on the length of the gene and reads count mapped to this gene. FPKM, which is the expected number of Fragments Per Kilobase of transcript sequence per Millions base pairs sequenced, considers the effect of sequencing depth and gene length for the reads count at the same time, and is currently the most commonly used method for estimating gene expression levels [107].

### Differential expression analysis

Prior to differential gene expression analysis, for each sequenced library, the read counts were adjusted by the edgeR program package through one scaling normalized factor. Differential expression analysis of two samples was performed using the DEGSeq R package (1.20.0). The P values were adjusted using the Benjamini & Hochberg method. Corrected P-value of 0.005 and log2 (Fold change) of 1 (for *de novo* assembly: P value was adjusted using q value. qValue < 0.005 & |log2(foldchange)| > 1 was set as the threshold for significant differential expression).

### Novel transcripts prediction

The Cufflinks v2.1.1 Reference Annotation Based Transcript (RABT) assembly method was used to construct and identify both known and novel transcripts from TopHat alignment results.

### Phylogenetic tree

Amino acid sequences of selected AOX, CCE, FAR and DES were aligned with MAFFT (E-INS-I parameter). Thereafter, PhyML 3.1 with WAG substitution model was used to construct a maximum likelihood phylogenetic tree using Bayesian analysis. The deduced protein sequences used in the phylogenetic tree are listed in Additional file 5. Finally, the trees were viewed and group edited with FigTree v1.4.2 (http://tree.bio.ed.ac.uk/software/figtree/).

### RNA extraction and cDNA synthesis

Total RNA was extracted using EasyPure RNA Kit (TransGen Biotech, Beijing, China) following the manufacturer’s instructions, in which DNase digestion was included to avoid the genomic DNA contamination. RNA quality was checked with a spectrophotometer (NanoDrop 2000, Thermo Fisher Scientific, USA). The single-stranded cDNA templates were synthesized from 1 μg total RNA from various tissue samples using the PrimeScriptRT Master Mix (TaKaRa, Dalian, China) at 42 °C for 1 h. The reaction was terminated by heating at 70 °C for 15 min.

### Quantitative real time PCR and data analysis

qPCRs were performed for each sample using an iCycle iQ (Bio-Rad, CA, USA) according to the minimum information for publication of quantitative Real-Time PCR Experiments [108]. Gene-specific primers were designed by Beacon Designer 7.6 (PREMIER Biosoft International, CA, USA) and are listed in Additional file 6: Table S3. The mRNA levels were measured in three technical replicates from each of the three biological replicates by qPCR using TransStart Tip Green qPCR SuperMix, as described by the manufacturer (TransGen Biotech, Beijing, China). The mRNA levels were quantified using ribosomal protein L8 (*RPL8*) as the reference gene [56]. Means and standard errors were calculated based on at least two biological replicates. Relative expression level of the mRNAs for genes was calculated according to the 2^−ΔΔCq^ method. Relative fold-changes in the different tissues were calculated and normalized based on the transcript levels in the bodies.

## Additional files

Additional file 1: Table S1. The genome location of novel genes. (XLS 1969 kb)
Additional file 2: Figure S1. Annotation summaries of DBM unigenes. (A) Species distribution of unigenes with the best hit annotation terms in the non-redundant (Nr) database. (B) Gene ontology (GO) classifications of DBM unigenes. (TIF 817 kb)
Additional file 3: Table S2. Information of the pheromone biosynthesis and degradation genes in DBM. (XLSX 4618 kb)
Additional file 4: Figure S2. Multiple sequence alignment of PBANr variants from different insect species. The seven predicted TM domains are lined. Location of the YXXΦ endosomal sorting motif is highlighted in red. Pluxy, *Plutella xylostella*, Bommo, *B. mori*, Helar, Psese, *Pseudaletia separate*, Helvi, *H. virescens*, Chisu, *Chilo suppressalis*, Ostnu, *Ostrinia nubilalis*, Helze, *Helicoverpa zea (ZIP 4167 kb)*

Additional file 5: The phylogenetic tree of sex pheromone gene sequences used in this study. (XLSX 26 kb)
Additional file 6: Table S3. Sequences of primers used in qPCR. (TXT 647 kb)
Additional file 7: cDNA sequences of assembled genes. (XLS 38124 kb)
Additional file 8: The data evaluation of *P. xylostella de novo* tissue transcriptomes. (DOCX 15 kb)
Additional file 9: Table S4. The detailed annotation information of *de novo* assembly. (RAR 9052 kb)

## Abbreviations

ACC: Acetyl CoA Carboxylase
ACT: Acetyltransferase
ACT: Acetyltransferase
AD: Alcohol dehydrogenase
AKR: Aldo-keto reductase
ALDH: Aldehyde dehydrogenase
AOX: Aldehyde oxidase
AR: Aldehyde reductase
CCE: Carboxyl/cholinesterase
cDNA: Complementary DNA
CYP: Cytochrome P450
DBM: Diamondback moth
DES: Desaturase
FAR: Fatty acyl-CoA reductases
FAS: Fatty acid synthase
FATP: Fatty Acid Transport Protein
FPKM: Fragments per kilobase per million mapped fragments
GO: Gene ontology
GST: Glutathione S-transferase
MDR: Medium-chain dehydrogenases/reductase
PBANr: Pheromone biosynthesis activating neuropeptide receptor
PBP: Pheromone binding protein
PR: Pheromone receptor
qPCR: Quantitative real time qPCR
SDR: Short chain dehydrogenases/reductase
UGT: UDP-glycosyltransferases
