# Supplementary figures and images for: A reference gene set for sex pheromone biosynthesis and degradation genes from the diamondback moth, Plutella xylostella, based on genome and transcriptome digital gene expression analyses
Source: BMC Genomics. 2017 Mar 1;18:219. doi: 10.1186/s12864-017-3592-y (PMC5333385; doi:10.1186/s12864-017-3592-y)

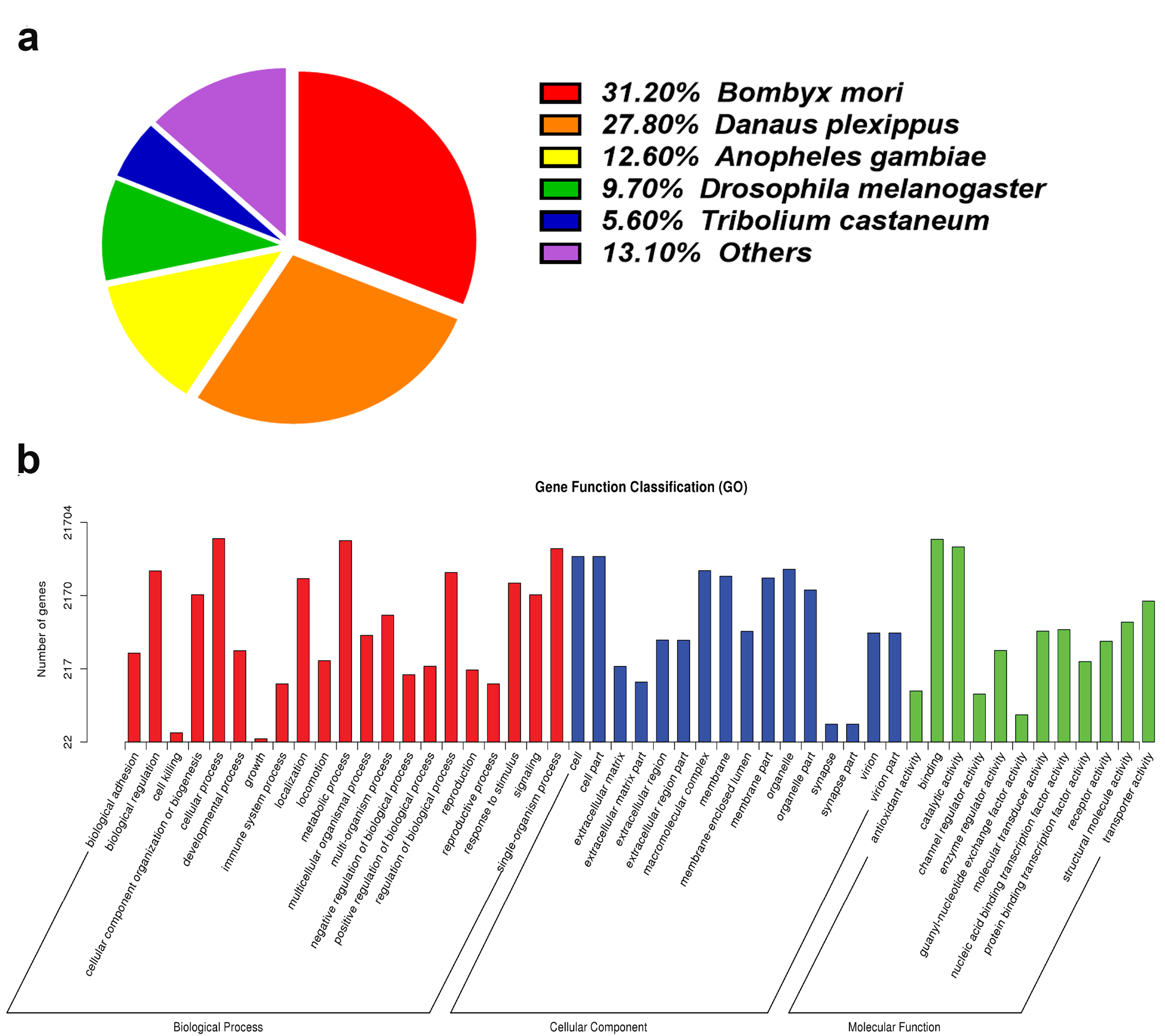

Supplement: Additional file 2: Figure S1. — Annotation summaries of DBM unigenes. (A) Species distribution of unigenes with the best hit annotation terms in the non-redundant (Nr) database. (B) Gene ontology (GO) classifications of DBM unigenes. (TIF 817 kb) [file 12864_2017_3592_MOESM2_ESM.tif]

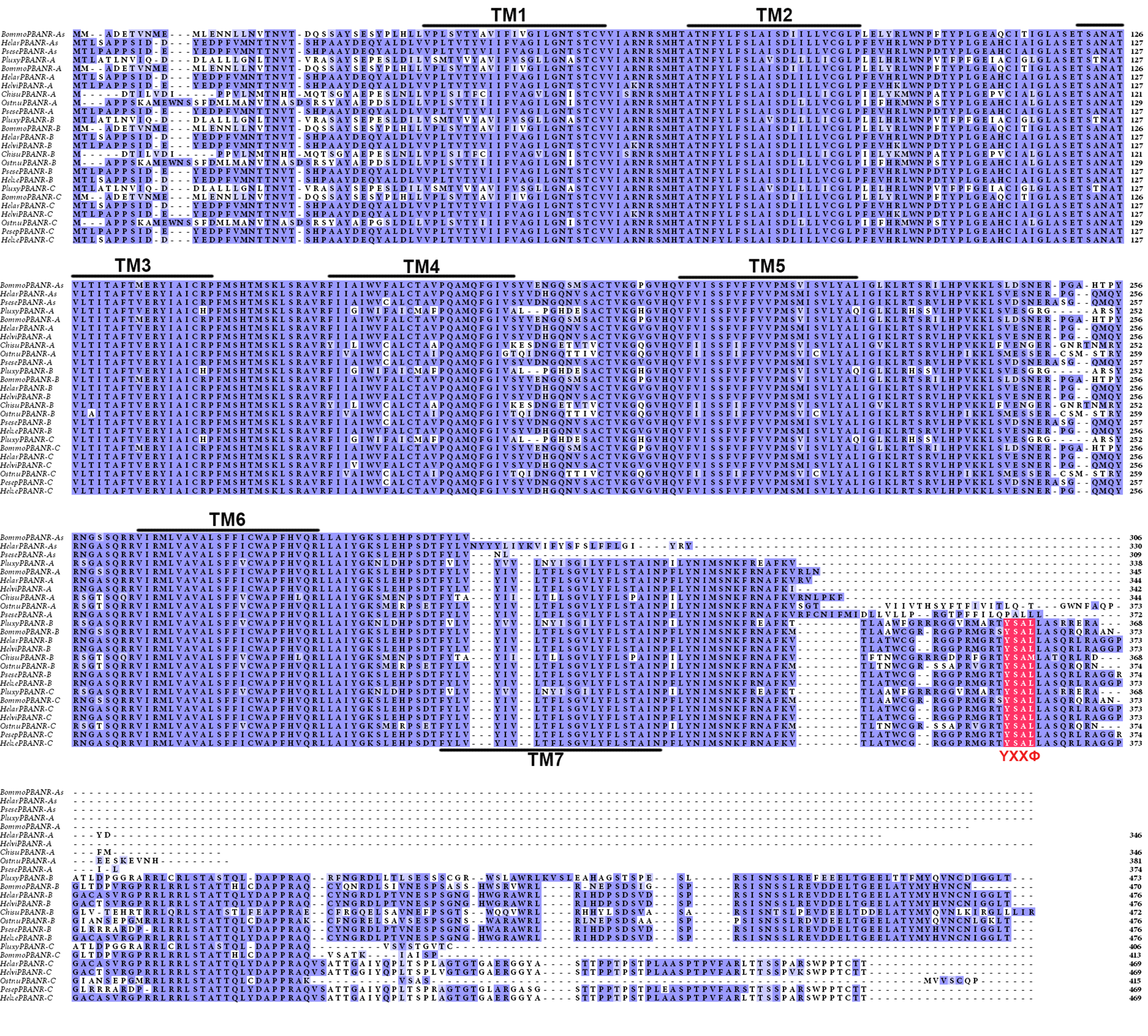

Supplement: Additional file 4: Figure S2. — Multiple sequence alignment of PBANr variants from different insect species. The seven predicted TM domains are lined. Location of the YXXΦ endosomal sorting motif is highlighted in red. Pluxy, Plutella xylostella, Bommo, B. mori, Helar, Psese, Pseudaletia separate, Helvi, H. virescens, Chisu, Chilo suppressalis, Ostnu, Ostrinia nubilalis, Helze, Helicoverpa zea (ZIP 4167 kb) [file 12864_2017_3592_MOESM4_ESM.zip › additional file 4/addtional file s4 figure s2.tif]
