# Supplementary material for: A reference gene set for sex pheromone biosynthesis and degradation genes from the diamondback moth, Plutella xylostella, based on genome and transcriptome digital gene expression analyses
Source: BMC Genomics. 2017 Mar 1;18:219. doi: 10.1186/s12864-017-3592-y (PMC5333385; doi:10.1186/s12864-017-3592-y)
Supplement: Additional file 8: — The data evaluation of P. xylostella de novo tissue transcriptomes. (DOCX 15 kb) [file 12864_2017_3592_MOESM8_ESM.docx]

| Sample | Raw Reads | Clean reads | Clean bases | Error (%) | Q20(%) | Q30 (%) | GC (%) |
| --- | --- | --- | --- | --- | --- | --- | --- |
| L_1 | 32212340 | 26719512 | 3.34G | 0.03 | 96.00 | 91.88 | 47.46 |
| L_2 | 32212340 | 26719512 | 3.34G | 0.04 | 93.38 | 87.67 | 47.45 |
| mA_1 | 30553607 | 25474013 | 3.18G | 0.03 | 95.77 | 91.53 | 47.26 |
| mA _2 | 30553607 | 25474013 | 3.18G | 0.04 | 93.77 | 88.30 | 47.26 |
| fA_1 | 32648481 | 27330117 | 3.42G | 0.03 | 96.08 | 92.05 | 45.98 |
| fA_2 | 32648481 | 27330117 | 3.42G | 0.04 | 93.74 | 88.25 | 45.97 |
| H_1 | 31142964 | 25558634 | 3.19G | 0.03 | 95.72 | 91.45 | 54.59 |
| H_2 | 31142964 | 25558634 | 3.19G | 0.04 | 93.06 | 87.02 | 54.58 |
| G_1 | 28405448 | 23878675 | 2.98G | 0.03 | 96.01 | 91.92 | 47.77 |
| G_2 | 28405448 | 23878675 | 2.98G | 0.04 | 93.41 | 87.78 | 47.76 |
| B_1 | 32719462 | 27303336 | 3.41G | 0.03 | 95.74 | 91.54 | 45.01 |
| B_2 | 32719462 | 27303336 | 3.41G | 0.04 | 93.73 | 88.30 | 45.02 |
